# Factors responsible for the emergence of arboviruses; strategies, challenges and limitations for their control

**DOI:** 10.1038/emi.2015.18

**Published:** 2015-03-25

**Authors:** Guodong Liang, Xiaoyan Gao, Ernest A Gould

**Affiliations:** 1 State Key Laboratory for Infectious Disease Prevention and Control, National Institute for Viral Disease Control and Prevention, Chinese Centre for Disease Control and Prevention, Beijing 102206, China; 2 Collaborative Innovation Centre for Diagnosis and Treatment of Infectious Diseases, Hangzhou 310003, Zhejiang province, China; 3 Aix Marseille University, IRD French Institute of Research for Development, EHESP French School of Public Health, EPV UMR_D 190 “Emergence des Pathologies Virales”, Marseille 13385, France; 4 Centre for Ecology & Hydrology, Oxford OX1 3SR, United Kingdom

**Keywords:** arbovirus, emergence

## Abstract

Slave trading of Africans to the Americas, during the 16th to the 19th century was responsible for the first recorded emergence in the New World of two arthropod-borne viruses (arboviruses), yellow fever virus and dengue virus. Many other arboviruses have since emerged from their sylvatic reservoirs and dispersed globally due to evolving factors that include anthropological behaviour, commercial transportation and land-remediation. Here, we outline some characteristics of these highly divergent arboviruses, including the variety of life cycles they have developed and the mechanisms by which they have adapted to evolving changes in habitat and host availability. We cite recent examples of virus emergence that exemplify how arboviruses have exploited the consequences of the modern human lifestyle. Using our current understanding of these viruses, we also attempt to demonstrate some of the limitations encountered in developing control strategies to reduce the impact of future emerging arbovirus diseases. Finally, we present recommendations for development by an international panel of experts reporting directly to World Health Organization, with the intention of providing internationally acceptable guidelines for improving emerging arbovirus disease control strategies. Success in these aims should alleviate the suffering and costs encountered during recent decades when arboviruses have emerged from their sylvatic environment.

## INTRODUCTION

Despite the announcement of the successful eradication of smallpox in 1979, the last case of rinderpest in 2008 and the current campaigns to eradicate poliomyelitis and measles through mass-immunization programmes, we still face the prospect of emerging or reemerging viral pathogens that exploit changing anthropological behavioural patterns. These include intravenous drug abuse, unregulated marketing of domestic and wild animals, expanding human population densities, increasing human mobility, and dispersion of livestock, arthropods and commercial goods via expanding transportation systems. Consequently, the World Health Organization concluded that acquired immune deficiency syndrome, tuberculosis, malaria, and neglected tropical diseases will remain challenges for the foreseeable future.^1^ Understandably, the high human fatality rates reported during the recent epidemics of Ebola, severe acute respiratory syndrome and Middle East respiratory syndrome have attracted high levels of publicity. However, many other RNA viruses have emerged or reemerged and dispersed globally despite being considered to be neglected diseases.^2,3^ Chikungunya virus (CHIKV), West Nile virus (WNV) and dengue virus (DENV) are three of a large number of neglected human pathogenic arthropod-borne viruses (arboviruses) whose combined figures for morbidity and mortality far exceed those for Ebola, severe acute respiratory syndrome and Middle East respiratory syndrome viruses. For instance, for DENV, the number of cases of dengue fever/hemorrhagic fever is between 300–400 million annually, of which an estimated 22 000 humans die.^4^ Moreover, in the New World, within 12 months of its introduction, CHIKV caused more than a million cases of chikungunya fever according to Pan American Health Organization/World Health Organization, with sequelae that include persistent arthralgia, rheumatoid arthritis and lifelong chronic pain.^5^ Likewise, within two months of its introduction, to Polynesia, the number of reported cases exceeded 40 000^6^ and is currently believed to be approaching 200000 cases. Alarmingly, this rapid dispersion and epidemicity of CHIKV (and DENV or Zika virus in Oceania) is now threatening Europe and parts of Asia through infected individuals returning from these newly endemic regions. This is an increasingly worrying trend. For example, in France, from 1 May to 30 November, 2014, 1492 suspected cases of dengue or chikungunya fever were reported.^7^ Accordingly, this review focuses on the emergence or reemergence of arboviruses and their requirements and limitations for controlling these viruses in the future.

## VARIETIES OF ARBOVIRUSES

Arboviruses are transmitted between arthropods (mosquitoes, ticks, sandflies, midges, bugs…) and vertebrates during the life cycle of the virus.^8^ Many arboviruses are zoonotic, i.e., transmissible from animals to humans.^9,10^ As far as we are aware, there are no confirmed examples of anthroponosis, i.e., transmission of arboviruses from humans to animals.^9,10^ The term arbovirus is not a taxonomic indicator; it describes their requirement for a vector in their transmission cycle.^11,12^ Humans and animals infected by arboviruses, may suffer diseases ranging from sub-clinical or mild through febrile to encephalitic or hemorrhagic with a significant proportion of fatalities. In contrast, arthropods infected by arboviruses do not show detectable signs of sickness, even though the virus may remain in the arthropod for life. As of 1992, 535 species belonging to 14 virus families were registered in the International Catalogue of Arboviruses.^12^ However, this estimate is continuously increasing as advances in virus isolation procedures and sequencing methods impact on virus studies. Whilst many current arboviruses do not appear to be human or animal pathogens, this large number of widely different and highly adaptable arboviruses provides an immense resource for the emergence of new pathogens in the future.

## ARBOVIRUS TRANSMISSION VECTORS

In addition to evolving strategies for long-term survival, arboviruses have an enormous choice of arthropod species potentially capable of being infected the predominant species of which appear to be mosquitoes and ticks. Approximately 300 types of mosquito can transmit arboviruses. *Aedes* and *Culex* mosquitoes are the species most frequently associated with arbovirus transmission (115 and 105 types of arbovirus, respectively).^12^ Ticks are also prevalent vectors, 116 different species are currently known to transmit arboviruses. In addition, 25 midge species have been shown to transmit arboviruses, mainly *Culicoides* (24 types) and *Lasiohelea*. Sandflies, blackflies, stinkbugs, lice, mites, gadfly, and bedbugs can also transmit arboviruses.^13^ This diversity of species and the wide distribution of these transmission vectors explain why arboviruses are so successful in dispersing globally via the mechanisms highlighted earlier.^10,11,12^ Arboviral diseases are primarily associated with specific vectors. However, many other arthropod species, in which viruses have been identified, may be involved in perpetuating the virus life cycle without having been associated with overt disease in humans or animals. For example, WNV is typically mosquito-borne but can be vectored by many different mosquito species and also by ticks and other arthropods.^9,10,12^ Moreover, Japanese encephalitis virus appears to be transmissible by *Culex*, *Anopheles*, and other mosquito species, as well as midges, sandflies and ticks. As a general rule, a specific arthropod species, is likely to predominate during an epidemic. However, if the availability of the vertebrate host, for example birds, becomes limited towards the end of the summer as the birds migrate to warmer countries, the vector might switch its preference to a different vertebrate host. This is consistent with the observation that outbreaks of West Nile fever/encephalitis in North America often appear to occur in the late summer, following peak incidence in birds, after they commence their migration to warmer regions.^14^ Alternatively, *Aedes aegypti* has adapted to the urban environment whereas *Aedes albopictus* occurs more commonly in semi-urban or rural areas. However, these are not hard and fast rules. For example, on the French island of La Reunion, where *Aedes aegypti* was not recognizably present, CHIKV was simultaneously epidemic in both rural and urban environments, and was primarily associated with *Aedes albopictus*.^15^

## WORLDWIDE DISTRIBUTION OF ARBOVIRUSES AND THEIR MOBILITY

A high proportion of arboviruses associated with human and animal disease circulate in tropical, and subtropical regions, where mosquitoes, and other flying insects, tend to be abundant. However, many arboviruses also circulate amongst wildlife species in temperate regions of the world. Despite the global distribution of viruses such as WNV, dengue virus, bluetongue virus and now CHIKV, most other arboviruses are generally endemic to specific regions of the world. For example, mosquito-borne Japanese encephalitis virus is prevalent in India, Central and Southeast Asia, largely due to the prevalence of highly competent *Culex tritaeniorhynchus* mosquito species and intensely farmed pigs which act as amplifying hosts for the virus and, importantly, the mosquitoes.^16,17^ In Southeast Asia, rice cultivation with enormous areas of paddy fields also attracts migratory birds and provides ideal breeding areas for the mosquitoes which transmit the virus to the birds, ensuring virus dispersion over large areas of Asia.^18^ In contrast, tick-borne encephalitis virus occurs mainly in northern temperate regions which are the primary habitats of *Ixodes* species ticks (forests etc.).^19^ Nevertheless, even within this relatively localized distribution of arboviruses, dispersion to distant locations occurs via animal or vector migration. As an example, Powassan virus, a close relative of tick-borne encephalitis virus, is found in Far East Asia and also in Canada.^19^ In contrast, Rift Valley fever virus (RVFV) was localized in Africa but in 1977, it was introduced to the Middle East, where it caused thousands of human infections, with an estimated 598 deaths, during a single epidemic.^20^

## ARBOVIRUSES AND RELATED INFECTIOUS DISEASES

More than 100 species of arbovirus that cause human/animal or zoonotic diseases have been identified.^12^ Four virus families, *Togaviridae, Flaviviridae, Bunyaviridae* and *Reoviridae*, contain most of the arboviruses that cause human/animal diseases.^12^ Arbovirus infections are not always clinically obvious and often resolve spontaneously after 1–2 weeks. However, some arboviral infections result in high fever, hemorrhage, meningitis, encephalitis, other serious clinical symptoms, and even death. Therefore, they cause a large social and economic burden. A summary listing the arboviruses associated with human diseases and their geographic distributions was published previously.^21^

## ARBOVIRUS SURVIVAL STRATEGIES

Arboviruses have evolved a wide variety of strategies to ensure their long-term success, dispersal and survival. They associate with specific arthropods and exhibit distribution characteristics that reflect the environmental preferences of these particular species. However, once an epidemic has run its course, the virus survives via its sylvatic life cycle which may involve a wide variety of species currently not identified. Alternatively, arboviruses can be maintained for months or maybe even years in mosquito eggs which remain dormant until the rainy season triggers hatching of the new, healthy but infected mosquito larvae.^21^ Eggs can also provide a long term reservoir for tick-borne arboviruses but in many cases the viruses exploit the extended life cycle of some tick species, surviving for years through the transstadial stages, reproducing at low levels.^21^ This long-term survival strategy is also enhanced by non-viraemic transmission during which infected and non-infected ticks co-feed on small animals in the forests. Non-viraemic transmission provides an efficient mechanism for transmission of the virus directly between ticks, without necessarily infecting the vertebrate host. Whilst co-feeding is taking place, virus infectivity in the tick salivary glands may increase by orders of magnitude presumably increasing virus transmission efficiency between the ticks.^22^ A similar non-viraemic co-feeding transmission process involving mosquitoes and blackflies, has also been described for WNV and vesicular stomatitis virus.^23^ Alternatively some insect-specific flaviviruses exist in the form of DNA when they infect insect cells.^24^ Diverse sequences have been discovered that appear to be related to insect-specific flaviviruses, amplified from mosquitoes, belonging to the culicine genera *Culex*, *Aedimorphus*, *Ochlerotatus* and/or *Stegomyia*. Many of these sequences may represent DNA integrations into mosquito genomes.^25,26,27,28,29,30,31,32,33,34^ Moreover, sRNAs related to WNV in apparently uninfected *Culex* mosquitoes have also been identified.^35^ Whilst there is no current evidence that such forms of DNA, provide a long-term arbovirus survival strategy, the similarity with other viruses that use DNA intermediates and episomal DNA should not be ignored. These and other diverse survival strategies provide safe havens for their long-term survival from which they can reemerge to cause epidemics amongst human and animal populations.

## DETERMINANTS OF ARBOVIRUS EMERGENCE

Changing anthropological behaviour, climate change and high mutation frequency are important determinants of arbovirus emergence. Arboviruses adapt readily to new susceptible hosts by alteration of receptor specificity, transmission efficiency, antigenicity, and ecological and environmental conditions. Humans, livestock and/or domestic animals are not an essential part of this arbovirus life cycle. Therefore, unlike, smallpox virus, measles virus or poliovirus, arbovirus disease control based on humans, livestock and/or domestic animals cannot eradicate the arbovirus. Consequently, the reservoir for arboviruses in wild species places a limitation on our ability to control disease emergence. For instance, CHIKV was a zoonotic arbovirus that cycled harmlessly between simians and mosquitoes in the African tropical forests causing localized outbreaks of polyarthralgia in humans. It also occasionally “escaped” to Asia gradually becoming zoonotic. However, prior to 2005, CHIKV was rarely an epidemic arbovirus until a mutation occurred in the gene encoding the surface protein of the African strain which increased its capacity to infect, reproduce and be transmitted by the striped Asian “Tiger” mosquito *Aedes albopictus*.^36^ Coincidentally this mosquito species has gradually dispersed westwards and CHIKV is now a major global human epidemic pathogen throughout Asia.^5^ Moreover, on 6 December 2013, it was reported to have crossed the Atlantic Ocean, reaching the French Caribbean island of Saint Martin from where it dispersed to the Americas.^37^ It also dispersed eastwards from southern Asia, reaching Polynesia by October 2014.^6^ Worryingly, CHIKV is now frequently being introduced into non-endemic Europe and northern Asia by incoming humans infected in the Americas and Polynesia. In the Americas, WNV, was first reported in New York in August 1999,^38^ following a hot and humid summer and many publications describe its emergence and dispersal.^21,39,40,41^ In contrast with CHIKV, the major determinant for the dispersal of WNV, throughout North America, over a period of five years was primarily birds and their associated *Culex* mosquito species.^36^ The dispersal and increasing epidemicity of dengue fever/dengue hemorrhagic fever which is confined to humans in the tropics, sub-tropics and southern temperate regions, can generally be attributed to human and *Aedes aegypti* population density increase during the past century, resulting from intensive urbanization and the influence of increased transportation of humans, commercial goods, livestock and major military movements across the oceans.^42^ On the other hand, RVFV is enzootic and confined to a wide range of animals, mosquitoes and sandflies throughout Africa and the Arabian Peninsula. Generally, the virus circulates without causing major disease outbreaks which usually arise following periods of rainfall when herded ruminants are introduced to RVFV-endemic areas. Two important factors are believed to have influenced the epidemiology of RVFV, firstly, major irrigation projects and secondly, the El Niño effect.^43^ In each case, cited above, a combination of two or more of the factors identified earlier have had an important influence on the appearance of these emerging arboviruses in new territories and/or old territories. Other recently emerging arboviruses, not included in this brief outline, include, Zika virus in Oceania, bluetongue virus and Schmallenberg virus in northern Europe and Bagaza virus in Spain.^44,45,46,47^

## STRATEGIES FOR ARBOVIRUS CONTROL

The concept of arthropod-borne disease transmission was born out of the studies of a physician, Josiah Clark,^48^ and 40 years later developed by Carlos Finlay^49^ who proposed mosquitoes as the agents for transmission of yellow fever. Subsequently, empirical methods, such as mosquito eradication, which was used very successfully in Cuba,^50^ and the development of a yellow fever vaccine has protected millions of humans from potentially fatal infection by yellow fever virus.^51,52,53^ Now, in the 21st century genetically engineered live attenuated vaccines can be manufactured within months, to protect humans against the ravages of pandemic influenza and other virus diseases. Moreover, a spectrum of antiviral molecules has been developed to treat humans dying from infection with human immunodeficiency virus and several antiviral drugs have also been developed against other viruses. Does this mean that if we develop vaccines and antiviral drugs to prevent or treat humans against infection by pathogenic arboviruses we will resolve the challenges associated with emerging arboviruses? Regrettably it is not that simple! It is a remarkable fact that in the future, because of their high mutation rates, many new pathogenic arboviruses will emerge even though they do not currently exist as epidemic strains in the sylvatic environment. It is also becoming clear, from early results of genome sequencing, that mosquitoes carry large numbers of known and unknown viruses that infect humans, primates, mammals, birds, insects, and plants.^53,54^ Therefore, should we attempt global eradication of arthropods? The answer is a definite no. This would have a catastrophic impact on the survival of many wildlife species, as first became apparent when dichlorodiphenyltrichloroethane was used widely as a general insect control agent rather than being precisely targeted to relevant mosquito species.^55^ However, implementation of temporary localized arthropod control measures during epidemics, for example in high density urbanized areas, can still play an important but transient role in reducing the impact on humans and animals of emerging arboviruses.

## CHALLENGES AND LIMITATIONS IMPOSED BY PATHOGENIC ARBOVIRUS

The breadth and depth of arbovirus surveillance differs regionally, and several areas lack surveillance altogether. There is also a lack of inter-disciplinary expertise on arbovirus diseases and understanding their vectors, and epidemiology. In addition, only a small number of arboviral diseases can be prevented using vaccines or specific antiviral drugs, and there are few validated diagnostic reagents, with which to monitor disease progress and control. Until such disease control reagents become available, the most effective alternative is to focus on practical procedures to reduce risks of exposure to arthropods.

## CONCLUSIONS AND RECOMMENDATIONS FOR THE DEVELOPMENT OF ARBOVIRUS CONTROL STRATEGIES

It is clear that we are now entering a period in virological discovery that will reveal a “Pandora's box” of new viruses with unique characteristics, which circulate in the ecosystem and will potentially lead to the evolution of novel pathogenic arboviruses. Future disease control strategies must therefore recognize this and be planned accordingly. What are the major objectives that need to be addressed if we are to win the war against these versatile viruses that appear to have an infinitesimal variety of strategies to confuse and disorganize our ability to bring them under control? (i) Develop vaccines to reduce the incidence of disease caused by known viruses; (ii) develop therapeutic drugs to treat clinical diseases caused by known viruses; (iii) develop unified vector-controlled strategies that will not unduly threaten the survival of wildlife species but locally will reduce the risk of disease in humans and animals; (iv) develop universal teaching/training courses to be taught worldwide, to provide cores of expertise to implement these policies; (v) encourage the strengthening of levels of cooperation between academia and drug and vaccine development companies; (vi) encourage the development of research programmes to understand the underlying mechanisms of arboviral pathogenicity, evolution, emergence and dispersal; (vii) develop, at the international level, public health measures to inform and educate citizens in local arboviral disease control measures, including monitoring and reporting; (viii) implement measures to improve monitoring procedures at borders, harbours, airports to reduce the influx of arthropods to new countries; (ix) develop unified public health strategies for arboviral disease control; (x) simplify the procedures for establishing safety and efficacy of antiviral drugs; (xi) establish an international committee of experts charged with the objective of reviewing global arthropod control strategies; (xii) develop and implement internationally acceptable and user-friendly guidelines for avoiding exposure to the different types of arthropod likely to carry human pathogens. This is undoubtedly an incomplete list of recommendations and probably some recommendations cannot easily be implemented. Nevertheless, the list provides the building blocks for a unified strategy on which to develop methods that will reduce the high morbidity and mortality rates due to human or animal arbovirus infections. Finally, we also have to recognise that arboviruses and arbovirus-related viruses infect more than just humans, invertebrates and land-based animals. They also infect plants, fish and marine animals. These relatively new discoveries also need to be accommodated if arboviral disease strategies are to be developed in a rational and unified manner. The lesson of the past is that human endeavour is at its best in the face of adversity. Let this be the mantra for arbovirus disease control in the future!
